# River zebrafish combine behavioral plasticity and generalized morphology with specialized sensory and metabolic physiology to survive in a challenging environment

**DOI:** 10.1038/s41598-023-42829-0

**Published:** 2023-09-29

**Authors:** Piyumika S. Suriyampola, José Jaime Zúñiga-Vega, Nishad Jayasundara, Jennifer Flores, Melissa Lopez, Anuradha Bhat, Emília P. Martins

**Affiliations:** 1 School of Life Sciences, Arizona State University, Tempe, AZ 85281 USA; 2 Departamento de Ecología y Recursos Naturales, Facultad de Ciencias, Universidad Nacional Autónoma de México, 04510 Mexico City, Mexico; 3 School of Marine Sciences, University of Maine, Orono, ME 04469 USA; 4 Department of Biological Sciences, Indian Institute of Science Education and Research-Kolkata, Mohanpur, 741246 India

**Keywords:** Ecology, Zoology, Climate sciences, Environmental sciences, Hydrology, Anatomy

## Abstract

Phenotypes that allow animals to detect, weather, and predict changes efficiently are essential for survival in fluctuating environments. Some phenotypes may remain specialized to suit an environment perfectly, while others become more plastic or generalized, shifting flexibly to match current context or adopting a form that can utilize a wide range of contexts. Here, we tested the differences in behavior, morphology, sensory and metabolic physiology between wild zebrafish (*Danio rerio*) in highly variable fast-flowing rivers and still-water sites. We found that river zebrafish moved at higher velocities than did still-water fish, had lower oxygen demands, and responded less vigorously to small changes in flow rate, as we might expect for fish that are well-suited to high-flow environments. River zebrafish also had less streamlined bodies and were more behaviorally plastic than were still-water zebrafish, both features that may make them better-suited to a transitional lifestyle. Our results suggest that zebrafish use distinct sensory mechanisms and metabolic physiology to reduce energetic costs of living in fast-flowing water while relying on morphology and behavior to create flexible solutions to a challenging habitat. Insights on animals’ reliance on traits with different outcomes provide a framework to better understand their survival in future environmental fluctuations.

## Introduction

Water flow is a key ecological parameter that can have multilevel impacts on phenotypes of aquatic organisms. Frequent strong flows can impose considerable energetic costs for swimmers^1–3^ and changes in flow can obstruct sensory systems through background noise^4^. Phenotypes are often flexible in variable environments^5,6^, and we might expect organisms in rivers to be plastic, changing behavior quickly as they move in and out of flowing water. Alternatively, river organisms may reduce the expense of reacting unnecessarily to constant change by responding sluggishly to small changes in water flow^7,8^. From this perspective, we might expect river organisms to exhibit general phenotypes that function well in a wide range of habitats at a low cost^9^. Insight into the strategies used by organisms living in fluctuating environments lays the foundation to better understand their survival in future environmental conditions. Here we measured the extent to which fish from rivers differ in behavioral, sensory, morphological, and metabolic traits in comparison to fish from still-water habitats.

Behavioral plasticity is one of the mechanisms through which aquatic organisms rapidly respond to the changes in flow conditions because flow can often impose important energetic constraints on locomotion. For example, many fish species tend to form large and more cohesive groups when in flowing water than in still water^1,2,10^. By forming cohesive, well-coordinated groups, animals gain hydrodynamic benefits under certain flow conditions^11^. Since, riverine species regularly experience rapid fluctuations in water flow, sometimes within seconds^12^, we might expect river fish to display a higher degree of plasticity, particularly in swimming behavior, than do fish inhabiting still-water habitats. For example, we might expect rapid adjustments in swimming velocity by river fish in response to changes in flow conditions. Alternatively, fish in fast-flowing rivers may maintain a low but stable swimming velocity by occupying less turbulent edges of rivers^13^ or by taking advantage of reduced flows behind physical structures in the environment^14^.

Water flow is the main physical property of aquatic systems that affects body form^15–17^. When living in a wide range of flow conditions, animals may benefit from morphologies that allow them to swim efficiently in different flow conditions. However, generalized morphologies that function across a broad range of contexts are sometimes sub-optimal and offer moderate performances compared to specialized morphologies that perform efficiently in a narrow range of niches^18,19^. Generally, streamlined bodies promote fast and prolonged swimming, and are ideal for swimming in uniformly flowing, fast currents^16,20^. Thus, we might expect river fish to display more streamlined bodies. However, streamlined body forms may not be cost-effective if flow conditions are irregular with intermittent turbulence and weak flow. Rivers exhibit a wide range of variation in water flow and turbulence^12,21^, and less streamlined, deeper bodies are better for reducing recoil and performing quick turns^16,22–24^.

Modifications in physiological mechanisms also play an important role in keeping up with environmental fluctuations. Sensory systems are a fundamental link between an animal’s environment and its physiology and behavior^25^. Certain modifications in sensory pathways may enable animals to sense specific changes in the environment and respond appropriately. For example, fish that respond behaviorally to changes in water flow may do so because they developed in fast-flowing water and harbor more sensory cells on the tail fin than do those that developed in slow-flowing water or lakes^26^. On the other hand, sensing every environmental change could be costly because neural systems may become overwhelmed with the continuous flow of information^8,27^, which could diminish focal attention and cognitive processes^7,28^. Thus, individuals in shifting environments may instead rely on sensory filtering or habituation to prevent information from swamping perception. Lake fish, in contrast, may either respond weakly to small changes in water flow due to the lack of exposure required to develop a highly sensitive lateral line system^26^, or respond strongly to water flow in a form of novelty effect^29^.

Differences in metabolic mechanisms may also allow organisms to respond more or less quickly to environmental change^30^. Metabolic rate is an important physiological influencer of swimming behavior because water flow directly affects locomotion by imposing additional energetic constraints^31,32^. Fish in fast-flowing water may possess higher metabolic rates^33^ due to the recruitment of more and faster muscle fibers^34,35^. However, constantly elevated metabolic rates are not always cost-effective. For example, small mammals in unpredictable environments tend to maintain low basal metabolism to offset energetic costs associated with environmental variability^36,37^. Fish may also reduce oxygen consumption by occupying the edges of turbulent rivers instead of open water^13^, swimming only to hold position^38^. By maintaining low oxygen demands, river fish may employ a generalist’s strategy and be able to survive in a wide range of flow conditions at a minimal cost. Lake fish, in contrast, may maintain low metabolic rates due to the low prerequisite to recruit faster muscle fibers^33^. However, novel contexts also elevate heart rate^39^ and thus, fish in still-water habitats may elevate metabolic rates when confronted with presence or changes in flow.

To measure differences in behavioral, morphological, sensory, and metabolic traits associated with flow, we used wild-caught zebrafish (*Danio rerio*) from four different sites. Zebrafish are small cyprinids native to parts of the Indian subcontinent^40^, and provide an outstanding model for this study as they occur in a wide range of habitats including still water, slow-flowing streams, and rapid rivers^10,41,42^. Zebrafish from one river site change their behavior when experiencing weak flows by forming less cohesive but more aggressive and active groups^43^. Here, we expand on these earlier results by comparing zebrafish from three additional sites that differ in flow conditions, and by reporting novel findings on morphometric, sensory, and metabolic, differences. Specifically, we asked whether zebrafish from two river sites differed from fish from two still-water sites in terms of swimming behavior, body shape, rheotaxis orientation towards a water flow, a measure of sensory sensitivity^44^, and tissue-specific and organismal bioenergetic metabolism.

## Results

### Zebrafish swam faster in flowing water, and river fish more rapidly adjusted swimming velocity than did fish from still-water habitats

**Figure 1:**
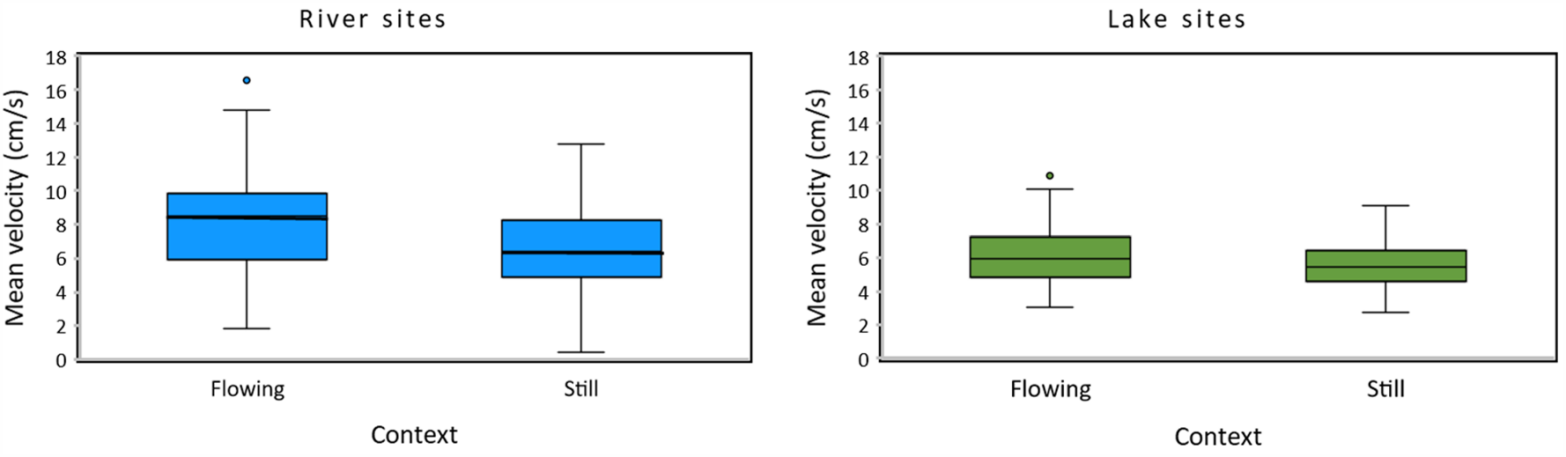
The average velocity of river (R1 and R2 sites combined) and still-water (L1 and L2 sites combined) zebrafish when tested in still and flowing contexts. Fish from river sites swam more quickly than did fish from still-water sites, and decreased velocity more dramatically when tested in still as compared to flowing water. Error bars represent 95% confidence intervals, the bottom and top of the box are the 25th and 75th percentiles, the line inside the box is the 50th percentile (median), and any outliers are shown as open circles.

Zebrafish swam faster when in flowing (7.0 ± 0.26 cm/s) than in still (6.2 ± 0.22 cm/s) water (Fig. 1), such that all three statistical models with strongest support included a term for treatment (flowing or still water assay context; Table 1). This difference was due primarily to a shift in velocity of zebrafish from the two river sites when measured in flowing vs. still water (from 8.1 ± 0.41 to 6.7 ± 0.37 cm/s; Fig. 1). Zebrafish from the two still-water lakes adjusted their swim velocity only slightly between the two treatment conditions (from 6.0 ± 0.23 to 5.7 ± 0.22 cm/s). Zebrafish from the two river sites also swam more quickly (7.3 ± 0.28 cm/s) than did zebrafish from the two still-water sites (5.8 ± 0.16 cm/s). As a result, we found that the best-fit model included an interaction (*b* = − 0.5 ± 0.41) between source habitat (river or still-water; fixed main effect: *b* = 2.0 ± 0.70) and treatment (flowing or still water assay context; fixed main effect: *b* = − 0.7 ± 0.29) as well as a global intercept (*b* = 6.6 ± 0.49) and random effects for population (nested within Habitat: s^2^ = 0.35) and group identity (the repeated-measures term: s^2^ = 1.34). However, a model including additive rather than interactive effects and a model including a single fixed effect for treatment alone fit equally well (the difference in Akaike Information Criteria scores adjusted for small sample sizes (ΔAICc) < 2), emphasizing the importance of flowing or still water during the assay itself (Table 1).

**Table 1:** The best-fit model based on Akaike information criteria adjusted for small sample sizes (AICc) predicted velocity as a function of an interaction between source Habitat (river or lake) and treatment (flowing or still water context), as well as fixed effects for both factors plus random effects for site (nested within habitat) and group (a repeated-measures term).

Model	AICc	ΔAICc	*w*	K
Treatment × habitat	870.6	0.0	0.47	7
Treatment + habitat	871.3	0.7	0.33	8
Treatment	872.3	1.7	0.20	6
Habitat	887.3	16.7	0.00	6
Null (intercept-only)	889.0	18.4	0.00	5

Similar models with an additive rather than an interaction effect, and with a single fixed effect for treatment also fit well.  + indicates an additive effect and  × indicates interaction. Models are listed according to their fit to the data (as indicated by AICc scores), from best to worst. *K* number of parameters, **Δ***AICc* difference in AICc scores between each model and the best-fitting model, *w* relative support for each model.

### River zebrafish were larger and less streamlined than were zebrafish from still water

**Figure 2:**
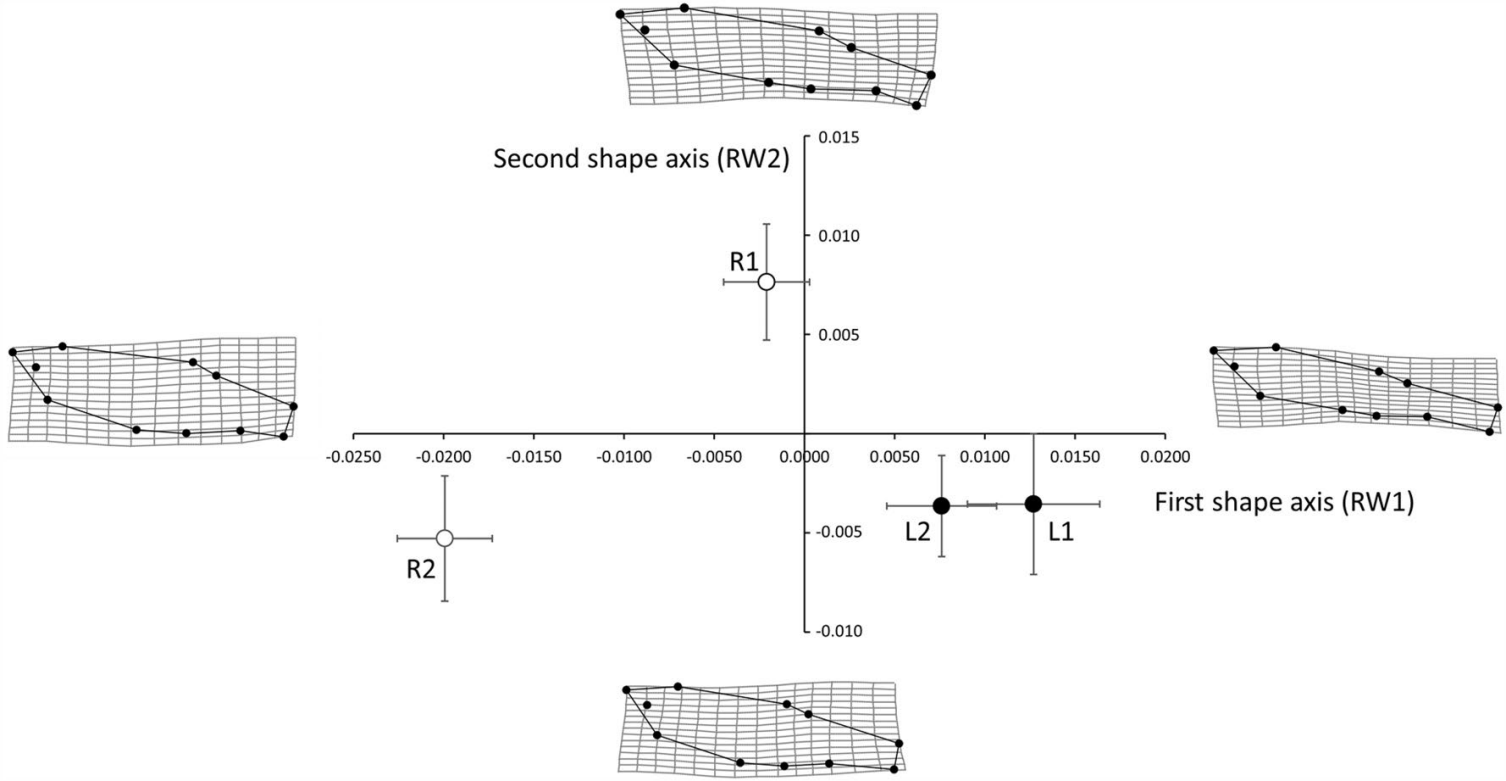
Shape variation of river (open circles: R1 and R2) and still-water (filled circles: L1 and L2) zebrafish. Zebrafish from still-water lakes were narrower in body with long, thin caudal peduncles than were zebrafish from rivers. Circles denote mean scores in the first (x = RW1) and second (y) = RW2 axes of shape variation (relative warps). Error bars represent 95% confidence intervals. Deformation grids depict deviations from the overall consensus shape representing the extremes of each axis, and are 2x-scaled to improve visualization of shape differences.

Zebrafish collected from rivers were less streamlined with shorter, deeper bodies and caudal peduncles than were fish from still-water lakes (Fig. 2). Fish from both still-water sites had larger positive values of the first Relative Warp (RW1, explaining 27% of total variation in body shape, x-axis in Fig. 2), corresponding to streamlined fish with narrow bodies and long, thin caudal peduncles, whereas zebrafish from river sites had negative values of RW1 indicating fish with deep, stocky bodies and short, thick, caudal peduncles (Fig. 2). In addition, zebrafish from the R1 site had larger positive values of RW2 (which explained 14% of the total variation in body shape), with dorsal fins set closer to the tail and longer anal fins than fish from the three other sites (negative values of RW2; Fig. 2). As a result, using either MANCOVA or univariate analyses, we found significant differences in relative warp scores between wild zebrafish collected from different source habitats (rivers vs. still water) and sites (nested within habitat; Table 2). Although total lengths were quite similar for fish from all four sites (mean = 2.2 cm, SE = 0.01), the effect of centroid size on relative warp scores was also statistically significant (Wilks’ Λ = 0.5, *P* < 0.001), reflecting larger centroids estimated for the deep-bodied fish from river sites.

**Table 2:** Results of multivariate and univariate analyses of variance examining variation in all relative warps (axes of shape variation) as well as separately in the first and second relative warps.

Response variable (s)	Explanatory variables	Test statistic	*P*
All relative warps	Site	Wilks’ Λ = 0.5	< 0.001
	Habitat type (river vs. lake)	Wilks’ Λ = 0.7	< 0.001
	Centroid size	Wilks’ Λ = 0.5	< 0.001
First relative warp (RW1)	Site	*F*_2,285_ = 5.8	< 0.001
	Habitat type (river vs. lake)	*F*_1,285_ = 9.3	0.003
	Centroid size	*F*_1,285_ = 43.5	0.002
Second relative warp (RW2)	Site	*F*_2,285_ = 4.8	0.009
	Habitat type (river vs. lake)	*F*_1,285_ = 5.1	0.025
	Centroid size	*F*_1,285_ = 0.1	0.765

### Zebrafish from rivers displayed weaker rheotaxis than did fish from still-water lakes

**Figure 3:**
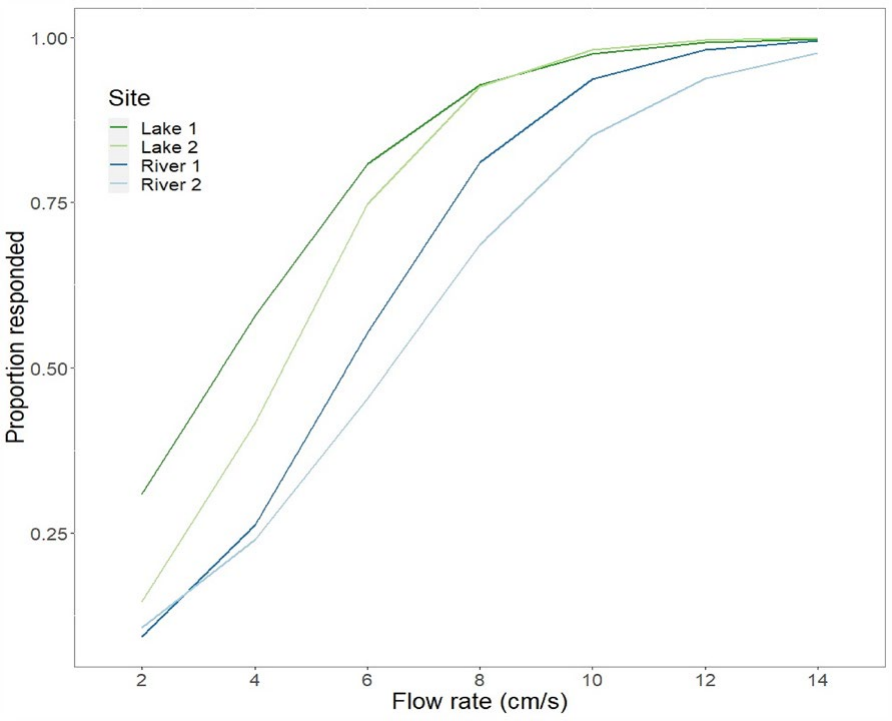
Logistic regression lines describing the observed differences in rheotaxis of zebrafish from river (blue lines) and still-water (green lines) sites to different flow rates. Overall, zebrafish from river sites oriented less towards flows (displayed weaker rheotaxis) than did zebrafish from still-water sites.

The odds that zebrafish collected from still-water lakes oriented to the flow was 9 times higher than the odds for zebrafish from rivers (slope = 2.2, SE = 0.77, e^2.2^ = 9), leading to a significant effect of source habitat (Z = 2.8, *P* < 0.01) in our repeated-measures logistic regression. For example, all of the fish from still-water lakes (100%) oriented towards the flow when the flow rate was 10 cm/s, but only 85% of the river fish oriented to the same flow. Zebrafish were more likely to orient toward the flow with increasing flow rate (Fig. 3), leading also to a significant effect of flow rate (Z = 7.5, *P* < 0.01). Individual differences were remarkably large (σ^2^ = 7.0, SE = 2.65) in comparison to differences between measures at different flow rates (σ^2^ = 3.2 × 10^–5^, SE = 5.7 × 10^–3^), between fish collected from different sites (σ^2^ = 4.7 × 10^–10^, SE = 2.2 × 10^–5^) or between fish collected from different source habitats (σ^2^ = 2.4 × 10^–7^, SE = 4.9 × 10^–4^).

### Zebrafish from a river site had lower basal heart metabolism and larger mitochondrial spare respiratory capacity

**Figure 4:**
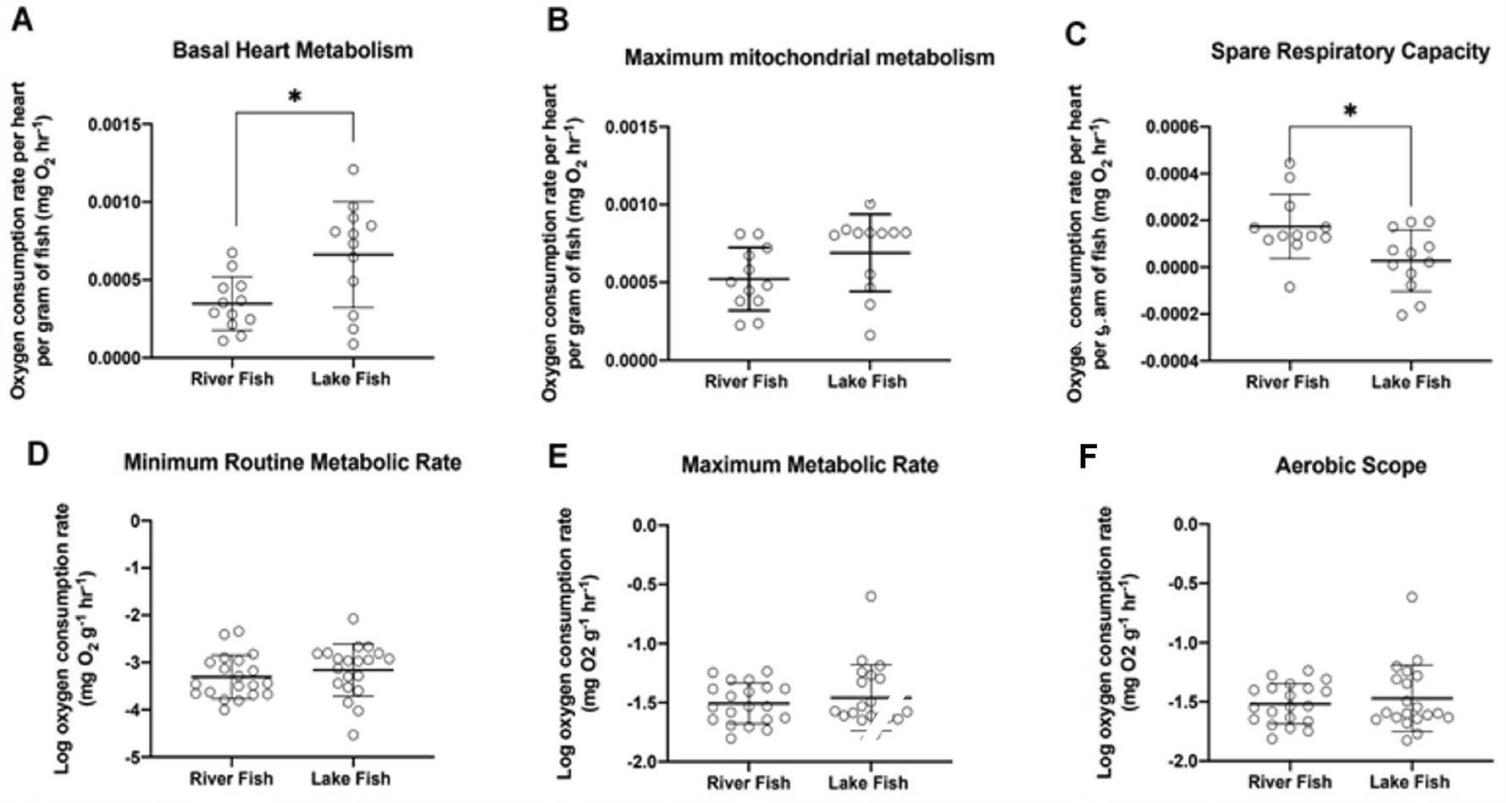
Cardiac (top) and whole-organism (bottom) metabolism measures for river (R1) and still-water (L1) fish. Basal (**A**) and maximal oxygen consumption rate (**B**) of an isolated zebrafish heart was higher in lake fish than in river fish. Spare mitochondrial capacity (**C**) was higher in river fish compared to the fish collected from still-water lakes. Although metabolic measures of whole organisms including minimum routine metabolic rate (**D**), maximum metabolic rate (**E**), and aerobic scope (**F**) were slightly higher in individual fish collected from the still-water site compared to river site, the differences between river and still-water fish were not statistically significant. Error bars represent 95% confidence intervals.

Oxygen consumption rates of isolated whole-heart tissues showed marked differences between fish from one river (R1) compared to one still-water (L1) site (Fig. 4A–C). Basal Heart Metabolism was significantly lower (0.0003 ± 0.00017 mg O_2_ per hour per heart per gram of fish) for river fish compared to still-water fish (0.0007 ± 0.00034 mg O_2_ per hour per heart per gram of fish; t_1, 16_ = 2.9, *P* = 0.01). FCCP-induced Maximum Mitochondrial Metabolism was relatively similar between fish from the two sites (t_1, 21_ = 1.8, *P* = 0.08). Consequently, mitochondrial Spare Respiratory Capacity (the difference between basal and maximal rates) was significantly lower (~ sixfold) in still-water fish than in river fish (Fig. 4C; t_1, 22_ = 2.7, *P* = 0.01). Some hearts of zebrafish collected from the still-water lake site actually operated at their maximal capacity under basal conditions, resulting in a decreased oxygen consumption rate upon FCCP-induced uncoupling (negative spare respiratory capacity).

Metabolic measures at the whole organism level followed the same patterns as the cardiac measures but did not differ significantly between river (R1) and still-water lake (L1) fish. For example, oxygen consumption during minimum routine metabolism (t_1, 38_ = 0.9, *P* = 0.37), maximum metabolism (t_1, 38_ = 0.7, *P* = 0.49), and aerobic scope (t_1, 38_ = 0.8, *P* = 0.42) were all slightly higher in still-water lake fish compared to river fish, but these differences were not statistically significant (Fig. 4D–F).

## Discussion

We found that river zebrafish swam faster, had lower oxygen demands, and were less likely to orient to flowing water than were fish collected from stagnant water, traits that are beneficial in fast-moving rivers^33^. River fish were less streamlined, with deep bodies that enhance rapid turning in small spaces rather than fast swimming^16^. Such morphological attributes may make river fish more suitable for the heterogenous conditions typical of flowing systems. Also, their velocity shifted dramatically when measured in flowing as opposed to still water: behavioral plasticity that would be especially useful for animals living in transitional habitats. In contrast, zebrafish collected from still-water sites were streamlined and highly sensitive to small changes in flow rates, but also slower swimmers with less efficient metabolisms. These findings offer insight into the mechanisms that allow animals to thrive in different aquatic environments.

In our study, river fish were especially plastic in terms of behavior and possessed morphologies well-suited to keep up with variable flow conditions in river habitats. River-dwelling species regularly experience intense and rapid fluctuations in water flow sometimes within seconds^12^. River fish in our study adjusted swimming velocity after being exposed to a small change in water flow for less than 24 h. Being able to adjust swimming velocity promptly allows fish to minimize costs while maintaining their position in strong currents, maximizing food capture, and intercepting chemical cues effectively^45,46^. Behavioral variability within populations is an adaptive strategy for coping with environmental fluctuation^47,48^. River fish, particularly in R2 population, displayed increased variability of swimming velocity, indicating the need to maintain an elevated level of plasticity. Flexibility may also arise through developmental exposure to certain environmental conditions that yield suitable, yet relatively, permanent traits such as specific morphs. Despite general predictions on fish body forms see^15,16,22^, we found that river zebrafish had less streamlined bodies with shorter caudal peduncles and longer anal fins compared to fish from still-water lakes. McGuigan et al.^33^ found similar results comparing the body shapes of wild-caught Australian rainbow fish from lakes and streams, but not in progeny from a common-garden experiment, suggesting that these differences may be the result of environmental rather than inherited effects. The deeper bodies and longer fins of river fish increase stability during turns^49,50^, as if designed for swimming in turbulence around the structurally complex habitats at the edges of streams, rather than in open water^12^. Further studies of microhabitat use by are needed to test this possibility and to identify environmental mechanisms that may guide development of fish shapes.

In other ways, it is more difficult to determine whether river fish are specialized to highly variable flows or to life in fast-flowing water. For example, river fish in this study responded only weakly to rheotaxis. Reduced sensitivity to environmental change suggests that river fish rely on “sensory filtering” or “habituation” mechanisms to prevent their sensory organs from being overloaded in constantly heavy flows^7^. Similarly, low resting metabolic rates may facilitate life in highly variable environments, as it does for small mammals maintaining homeostasis^36,51,52^. Similarly, low basal cardiac metabolism and higher aerobic scopes may enable river zebrafish to allocate more oxygen for muscles and to maintain high swimming speeds in fast-flowing water more efficiently^37^. We need additional research to tease apart phenotypic aspects that contribute to high functional versatility across a range of flow conditions and those that are tailored to a single specialized context^18,19^.

Although zebrafish from still-water lakes displayed low swimming velocity and flexibility, they had more robust rheotactic responses to weak flow rates, maintained higher metabolic rates, and were more streamlined in body shape. The relatively strong rheotaxis that we observed in still-water fish could be a baseline response to tactile cues or the result of a novelty effect^29^. Although zebrafish likely experience fewer changes in flow conditions in lentic habitats than in rivers, seasonal changes in water flow due to monsoons may be a particularly important environmental cue^53^. Similarly, still-water fish may have to maintain elevated oxygen consumption rates due to the low levels of dissolved oxygen in their environment or high risk of predation^54^, both of which are true for the still-water sites in the current study^10^. The capacity to increase cardiac output is critical to sustaining aerobic performance under increasing swimming activity and enhances cardiac mitochondrial performance^55–57^. High resting metabolism may also explain why still-water lake zebrafish in our study did not swim as quickly as did those from rivers, despite their streamlined body forms.

The distinct differences in behavior, morphology, sensory, and metabolic physiology that we observed between the river and still-water fish indicate that organisms integrate multiple traits to respond effectively to the challenges of living in fluctuating environments. Sensory mechanisms and metabolic physiology reduced energetic costs of living in fast-flowing water, while morphology and behavior created flexible solutions to the challenges of a transitional lifestyle in rivers. Generalization of traits such as behavior and morphology may provide organisms the flexibility to survive in changing environments but the specialization of other traits may limit their ability to keep up with environmental change. Thus, investigating variable outcomes of associated traits is important to better understand how different mechanisms will enable organisms to respond to future environmental changes.

## Methods

### Study subjects and maintenance

We collected zebrafish from two river and two still-water sites in India and exported them to the United States for laboratory experiments. The first river site (R1), was the “FM” site at the Torsa River in north-eastern India, described in Suriyampola et al.^58^. At this site, zebrafish were found in both still and rapidly flowing water (14 cm/s). Suriyampola et al.^43^ report different behavioral measures of zebrafish from this site. The second river site (R2) was at the Brahmani River in Odisha with similarly variable water flow (up to 17 cm/s). For the still-water comparisons, we collected zebrafish from two stagnant irrigation canals with little vegetation cover in West Bengal: L1 site is “SN” from Suriyampola et al.^10^ and the L2 site is “KB” from Roy and Bhat^59^. The R1 site is separated from L1 and L2 sites by about 600 km, whereas the L1 and L2 sites are separated from each other by about 180 km. All three sites fall into the Ganges/Brahmaputra group and are likely subject to considerable gene flow^60^. The R2 river site is geographically distinct (about 600 km away from L1 & L2 sites and 900 km away from R1 site) and located in the Brahmani/Baitarani river basin^61^, and thus, likely contains fish that are genetically distinct from those collected at the other three sites^60^.

In the lab, we housed zebrafish from each site separately in mixed-sex groups with a 14:10 h light: dark cycle, water temperature at 28 ± 1 °C, and daily feedings of commercial flake food (Tetramin Tropical). We began the experiment after fish acclimated to our laboratory conditions for two months, thereby ensuring also that all the zebrafish were adults and in good health. To ensure minimum handling stress, we used different groups of fish for behavioral, morphological, sensory, and metabolic assessments.

### Swimming velocity

To measure plasticity in swimming velocity in still- and flowing-water contexts, we followed the procedures, testing arenas, and experimental design described in Suriyampola et al.^43^ for fish from the R1 site, repeating assays to determine swimming velocity of fish from this and the three other sites. In brief, we formed mixed-sex groups of 6 adult fish (3 males and 3 females in each) and placed half of the groups in aquaria (20.8 L) with flowing water and the other half in aquaria without water flow. To create water flow, we turned on an aquarium filter that generated a gentle unidirectional flow of 4 cm/s. After about 20 h of acclimation (about 1 h after lights came on the following morning), we video-recorded each group of fish engaged in undisturbed behavior for a total of 3 min using webcams (Logitech® c525 HD) at 30 frames/s. At the end of the trial, we altered the water flow in each test arena (turning filters on or off), left the groups to acclimate to the new testing conditions for another 20 h, and repeated behavioral recording the following day. We tested 33 groups of R1 fish, 19 groups of R2 fish, 22 groups of L1 fish, and 31 groups of L2 fish, for a total of 630 fish.

We used EthoVision XT10 (Noldus Information Technology, 2013) software to track zebrafish from video recordings automatically. The software determined the x and y coordinates of each fish every 0.03 s (1790 moments/min) and used those coordinates to calculate the velocity of fish. Ethovision tracked all 6 fish well in our test arenas and dropped an average of only 3.2% of the 5370 moments in each trial because the software was unable to locate one or more of the fish. We did not see any differences between experimental treatments or source sites in this proportion. We estimated ***Velocity*** as the average velocity of 6 fish during 3 min.

To compare river and still-water fish in terms of swimming behavior, we used AIC model-fitting procedures to predict Velocity from source habitat (river or still-water) and treatment condition (flowing or still), with an interaction between habitat and treatment indicating the degree of behavioral plasticity. Our models considered also main effects of source habitat and treatment, plus a factor indicating source site (1 or 2, nested within habitat). We used the *lmer* function in the ‘lme4’ package^62^ of R^63^ to estimate model parameters and to compare a full model with simpler models, for example, that did not include the interaction term.

### Morphology

**Figure 5:**
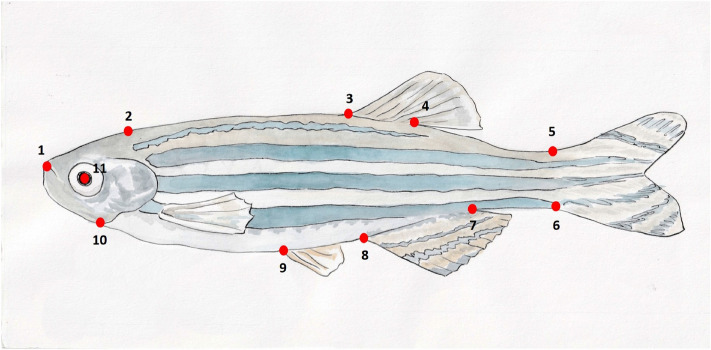
Locations of 11 anatomical landmarks used for morphometric analysis: (1) tip of the snout, (2) indentation at the posterodorsal end of head, (3) anterior insertion of the dorsal fin, (4) posterior insertion of the dorsal fin, (5) dorsal insertion of the caudal fin, (6) ventral insertion of the caudal fin, (7) posterior insertion of the anal fin, (8) anterior insertion of the anal fin, (9) anterior insertion of the pelvic fin, (10) opening of the operculum, and (11) center of the eye.

We used morphometrics to determine whether and how fish from rivers and still-water sites differed in size and shape. We photographed 98 fish from the R1 site, 56 fish from the R2 site, 65 from the L1 site, and 71 from the L2 site, placing each fish in a thin, custom-made, photography tank (two sheets of Plexiglas separated by plastic tubing), and taking lateral photographs with a Nikon D5000 camera and AF-P DX NIKKOR 18-55 mm f/3.5–5.6G VR lens. The number of males and females that we photographed was relatively similar in all four sites to avoid bias caused by potential differences in body shape between sexes (R1: 52 females and 46 males, R2: 28 females and 28 males, L1: 34 females and 31 males, L2: 42 females and 29 males). However, for our morphometric analysis we pooled data from both sexes because zebrafish lack marked sexual dimorphism^64^. Using tpsDIG2^65^, we scored 11 anatomical landmarks on the lateral profile of each fish (Fig. 5). Based on these landmarks, we computed a set of shape variables for each individual using the thin-plate spline approach^66^ as implemented in tpsRELW32^67^. In brief, we calculated two different measures of shape variation for each individual. First, we calculated a set of uniform shape components, which are geometrically uniform changes in shape across the entire body of the fish (i.e. overall increases in width or length with respect to an average or consensus shape). Second, we calculated a set of non-uniform shape components (‘partial warps’), which are non-uniform changes in the position of a subset of landmarks with respect to other landmarks^66^.

We then applied a Principal Components Analysis to both uniform and non-uniform shape components to obtain relative warps, which are orthogonal axes of shape variation. These relative warps are directly interpretable because their scores represent a summary of how the shape of each individual deviates from the average (consensus) shape among all individuals and across all sites. The thin-plate spline approach allowed us to visualize relative warp scores in deformation grids^66^. In addition, we calculated centroid size (a geometric measure of overall body size) for each fish as the square-root of the sum of squared distances from each landmark to their arithmetic center.

To test for differences in body shape between fish from rivers and still-water lakes, we conducted a nested multivariate analysis of covariance (nested MANCOVA) in which we used the relative warp scores as response variables, source habitat (river or still-water lake), and site (nested within habitat) as predictor variables. We also used centroid size as a covariate to account for differences in body size. Then, we examined the first two relative warps (explaining most of the variation in body shape) in nested univariate analyses of covariance (ANCOVA) to visualize major differences in shape between fish from river and still-water sites. In these ANCOVAs, we again considered the effects of source habitat, site (nested within habitat), and centroid size. We conducted these statistical analyses using Statistica version 10.0 (StatSoft Inc.).

### Sensory behavior (rheotaxis)

We used a 170 ml Blazka-type swim tunnel from Loligo® Systems (Denmark) to measure rheotaxis of individual fish, calibrating flow velocities using a Flow Tracking system (DPTV). We measured 17–20 fish from each site (19 R1 fish, 20 R2 fish, 17 L1 fish, and 19 L2 fish, for a total of 75 fish), moving each fish individually to the swim tunnel apparatus, and letting them acclimate for 5 min before exposing them to each of seven flow rates (30 s each in random order with 30 s intervals in between). The seven flow rates ranged from 2 to 14 cm/s, matching flow conditions commonly experienced by wild zebrafish see^10,40^. We video-recorded these trials using an uEye camera (uEye, Imaging Development Systems, Germany) at 30 frames/s mounted on the side of the swim tunnel, and later scored fish that faced the current throughout the 30 s as exhibiting ***Rheotaxis***. If the fish turned back and forth, or turned away from the water current, then we recorded an absence of rheotaxis.

To determine whether river and still-water fish differed in terms of Rheotaxis, we fit a nested, repeated-measures, logistic regression model to estimate the effect of source habitat (river or still-water, with site nested as a random effect within habitat) on the presence/absence of rheotaxis. Our model included also a repeated-measures factor because we measured the response of each fish at seven flow rates. We used the *glmer* function in the *lme4* package^62^ of R^63^ to estimate parameters.

### Cellular and whole-organism metabolism

To test for differences in cellular metabolism of fish from rivers and still-water lakes, we compared cardiac-tissue-specific oxygen consumption rates as a proxy for cardiac metabolism^57^, using fish only from R1 and L1 sites (n = 12 each). As described in Jayasundara et al.^57^, we excised whole hearts from euthanized fish, weighed, and washed them in a Ringer’s solution, and measured oxygen consumption rates using the XFe96 Extracellular Flux Analyzer (Agilent Technologies, CA). Euthanization was performed by cervical dislocation after anesthetizing each fish in ice water. We first measured ***basal heart metabolism***, and then injected the hearts with 0.002 mM of mitochondrial uncoupler FCCP (carbonyl cyanide4-(trifluoromethoxy)phenylhydrazone, Sigma-Aldrich, CAS370-86-5) to measure ***maximum mitochondrial metabolism***. Finally, we calculated ***spare respiratory capacity*** as the difference between Basal Heart Metabolism and FCCP-induced Maximum Mitochondrial Metabolism. We normalized these oxygen consumption rates by fish bodyweight (measure/fish-heart/gm of fish) and tested for differences between fish from the two sites in terms of Basal, Maximum, and Spare Capacities using Welch’s two-tailed *t* tests in R^63^.

Similarly, we measured whole-organism metabolism of 20 fish each from R1 and L1 sites. Here, we used the Loligo swim tunnel, submerging it in a 20-L buffering tank with a DAQ-M control device and respirometer using a fiber-optic oxygen instrument, and collecting data with AutoResp™ 1 software, (Loligo Systems, Tjele, Denmark). We tested fish individually at 28 °C and did not feed them for 24 h prior to the experiment. After moving individual fish to the swim tunnel, we waited 1 h for acclimation, and then recorded four dissolved oxygen levels in 20-min loops (a 10-min measuring phase followed by a 10-min flushing phase). The lowest oxygen consumption reading was our measure of ***minimum routine metabolic rate (MRMR)***. For ***maximum metabolic rate (MMR)***, we slowly increased the velocity to 10 body length/s and let the propeller run at this speed for 10 min or until the fish collapsed for 2 consecutive seconds. Upon fish collapse, we lowered the propeller speed to 1 cm/s (0.5 body lengths/s) and recorded oxygen consumption during roughly 5 min, estimating metabolic rate for each 1-min period and choosing the largest reading as the Maximum Metabolic Rate^57^. We calculated the ***aerobic scope (AS)*** as the difference between maximum metabolic rate and minimum routine metabolic rate. Again, we normalized these oxygen consumption rates by fish bodyweight (measure/gm of fish).

To test whether whole-organism metabolism differs between fish from rivers and still-water lakes, we fit an ANOVA model, testing the effect of site (river R1 or lake L1) on whole-organism metabolic measures (MRMR, MMR, and AS) using the base *aov* function in R^63^, and checking residuals to confirm that we did not violate the usual ANOVA assumptions of homoscedasticity and normality.

### Ethical note

Zebrafish research in this study was carried out in accordance with ARRIVE guidelines. All methods were in compliant with relevant guidelines and regulations and were approved by the Institutional Animal Care and Use Committees (IACUC) of Arizona State University (protocol number 20-1742R) and the University of Maine (protocol number A2017-05-04).

## Data Availability

The data that support the findings of this study will be openly available in figshare data reporsitory upon acceptance (https://figshare.com/s/5a843bc28c5af558f50b).
